# Mapping Prevalence, Diagnostics, and Evidence Gaps of Cryptosporidium in Southeast Asia Across Human, Animal, and Environmental Domains: Protocol for a One Health Scoping Review

**DOI:** 10.2196/89819

**Published:** 2026-06-19

**Authors:** Elad I Stotland, John Barlow, Andrea J Etter

**Affiliations:** 1 Department of Animal and Veterinary Sciences, College of Agriculture and Life Sciences, University of Vermont, Burlington, VT, United States; 2 Department of Nutrition and Food Sciences, College of Agriculture and Life Sciences, University of Vermont, 353 MLS Carrigan Wing, 109 Carrigan Drive, Burlington, VT, 05405, United States, 1 802-656-0541

**Keywords:** Cryptosporidium, cryptosporidiosis, Southeast Asia, ASEAN, One Health, zoonosis, waterborne disease, environmental surveillance, scoping review, diagnostic methods, Association of Southeast Asian Nations

## Abstract

**Background:**

*Cryptosporidium* is a waterborne and zoonotic protozoan parasite that causes cryptosporidiosis, a diarrheal disease that disproportionately affects young children and immunocompromised individuals in low- and middle-income settings. In Southeast Asia, ecological diversity, agricultural intensification, and uneven sanitation infrastructure create overlapping transmission pathways across human, animal, and environmental domains. Despite a growing body of regional literature, the structure of the evidence base and its utility for surveillance and control have not been comprehensively evaluated using an integrated One Health lens.

**Objective:**

This protocol outlines the methodology for a scoping review mapping the published literature on *Cryptosporidium* in Southeast Asia, characterizing the distribution of evidence on prevalence, diagnostic methods, species and genotype diversity, and environmental, food-related, and socioeconomic determinants of transmission across human, animal, and environmental domains.

**Methods:**

The review follows the Arksey and O’Malley framework, refined by Levac and colleagues and the Joanna Briggs Institute, and is reported in accordance with the Preferred Reporting Items for Systematic Reviews and Meta-Analyses Extension for Scoping Reviews (PRISMA-ScR). Five databases were searched from inception through September 30, 2024: PubMed, Embase, CABI Digital Library, Cochrane Library, and Index Medicus for the South-East Asia Region (IMSEAR), supplemented by backward citation tracking and targeted searches of organizational websites. Studies were included if they reported primary data on *Cryptosporidium* in human, animal, or environmental samples collected from one or more of the eleven Southeast Asian countries and addressed prevalence, diagnostic methods, or transmission risk factors. Reviews, editorials, letters, conference abstracts, and non-English publications were excluded. No formal risk-of-bias assessment was conducted, consistent with scoping review methodology. Data were organized thematically and synthesized descriptively, with findings presented across One Health domains using tables, figures, and maps generated in RStudio (version 2023.09.1+494).

**Results:**

Database searches retrieved 889 records before deduplication. After removing 177 duplicates, 711 unique records underwent title and abstract screening. Of 333 full-text articles assessed for eligibility, 176 studies were included in the final synthesis, representing nine of eleven Southeast Asian countries and spanning 1985 to 2024. Data extraction and analysis are complete. The manuscript reporting the full findings is being prepared for submission to a peer-reviewed journal.

**Conclusions:**

This scoping review provides a comprehensive cross-domain mapping of evidence on *Cryptosporidium* in Southeast Asia. The findings are expected to identify structural gaps in the regional evidence base, characterize diagnostic heterogeneity and its implications for surveillance utility, and support the development of integrated One Health surveillance strategies in the region.

## Introduction

*Cryptosporidium* is an apicomplexan protozoan parasite of global public health importance that causes cryptosporidiosis, a diarrheal disease that primarily affects young children, immunocompromised individuals, and populations with limited access to safe water and sanitation [1-3]. The parasite spreads via the fecal-oral route, with oocysts shed by infected humans and animals contaminating water, food, and the environment, where they persist because they resist standard chlorine disinfection [1,2,4]. The Global Enteric Multicenter Study (GEMs) identified *Cryptosporidium* as one of the four leading causes of moderate-to-severe diarrhea among children under five in low- and middle-income countries. Subsequent analyses estimated that approximately 202,000 deaths annually are attributable to cryptosporidiosis worldwide [5-7]. Worldwide, *Cryptosporidium* accounts for most protozoan-related waterborne outbreaks, with more than 1200 incidents recorded between 2004 and 2016 [8,9].

Effective control of *Cryptosporidium* requires identifying the transmission source—contaminated water, infected animals, or human-to-human spread—because each pathway requires a different intervention [1,2]. At least 40 species are recognized, infecting a broad range of vertebrate hosts: *C. hominis* and *C. parvum* account for over 90% of human infections globally. *C. parvum* circulates among livestock, wildlife, and humans; contaminated water and food serve as transmission bridges between animal reservoirs and human populations [10,11]. Yet, most *Cryptosporidium* research has been conducted in disciplinary silos—human clinical studies, veterinary surveys, and environmental monitoring are rarely integrated within a single investigative framework—leaving the relative contribution of each transmission pathway empirically uncharacterized in most settings [1,2]. Addressing this complexity requires a One Health approach, which recognizes that human, animal, and environmental health are interdependent and that effective disease prevention requires integrated, multisectoral surveillance and intervention [1,12].

Southeast Asia is a high-priority region for this problem. The region comprises eleven countries with highly variable ecological conditions, agricultural systems, and public health infrastructure, united by rapid economic development, expanding smallholder livestock production, and persistent inequalities in access to water and sanitation [12,13]. These conditions create overlapping exposure pathways—contaminated surface water used for irrigation and drinking, close human-animal contact in peridomestic and farm settings, and consumption of raw or minimally processed produce—that cannot be adequately characterized by studies examining any single exposure domain [1,4]. Numerous studies have documented *Cryptosporidium* prevalence in human populations, including children [14,15], individuals living with HIV/AIDS [16,17], and rural communities [18,19], as well as in livestock [20,21], companion animals [22,23], and wildlife [24], underscoring the parasite’s zoonotic potential. Environmental sampling has further revealed contamination of surface water, wastewater, and food sources [25-27], indicating that the environment plays a role in sustaining transmission cycles across the region. A recent review identified Southeast Asia as the Asian subregion of highest *Cryptosporidium* concern across human, water, and food sources, yet that analysis was limited to quantitative outcomes and did not examine diagnostic heterogeneity, species distribution, evidence on risk factors, or One Health integration across the regional literature [28]. The evidence base needed to act on that concern does not currently exist in usable form.

Despite the volume of regional literature, three structural features prevent the translation of available data into surveillance and control decisions. First, diagnostic heterogeneity is pervasive; microscopy-only studies, which cannot distinguish *Cryptosporidium* species or identify transmission sources, remain common across the region, and genotyping-based studies are not epidemiologically comparable to microscopy-only studies, yet both are routinely aggregated in regional analyses [28,29]. Second, geographic coverage is severely uneven, with research concentrated in a small number of countries, leaving most of the region’s epidemiological profile uncharacterized [13,28]. Third, cross-domain integration is rare; most studies examine a single host population in isolation, making it impossible to trace transmission pathways across the human-animal-environment interface that defines risk in smallholder agricultural settings [1,28]. Together, these features suggest that the current literature may be insufficient to support zoonotic attribution, environmental source prioritization, or risk-based intervention targeting across Southeast Asia — a question this review is designed to address systematically.

This scoping review protocol addresses these gaps by systematically mapping the published literature on *Cryptosporidium* from Southeast Asia across human, animal, and environmental domains. The review is guided by the following research questions: (1) What is the reported prevalence of *Cryptosporidium* across human, animal, and environmental domains in Southeast Asian countries? (2) What diagnostic methods have been used in the regional literature, and how does diagnostic heterogeneity affect species-level resolution and transmission inference? (3) To what extent do studies integrate evidence across human, animal, and environmental domains within a One Health framework? (4) What environmental, food-related, and socioeconomic risk factors have been quantitatively assessed in association with *Cryptosporidium* infection or detection in the region?

## Methods

### Study Design and Framework

This scoping review follows the methodological framework proposed by Arksey and O’Malley [30], with enhancements by Levac et al [31], and guidance from the Joanna Briggs Institute (JBI) [32]. Reporting adheres to the Preferred Reporting Items for Systematic Reviews and Meta-Analyses Extension for Scoping Reviews (PRISMA-ScR) [33], and the search strategy is reported in accordance with the Preferred Reporting Items for Systematic Reviews and Meta-Analyses extension for Reporting of Systematic Searches (PRISMA-S) [34]. The completed PRISMA-ScR checklist is provided as a supplementary file. The protocol was registered prospectively on the Open Science Framework (osf.io/qv27y)

### Objectives

This review aimed to map the existing literature on *Cryptosporidium* in Southeast Asia and was guided by the following research questions: (1) What is the reported prevalence of *Cryptosporidium* across human, animal, and environmental domains in Southeast Asian countries? (2) What diagnostic methods have been used across the regional literature, and how does diagnostic heterogeneity affect species-level resolution and transmission inference? (3) To what extent do studies integrate evidence across human, animal, and environmental domains within a One Health framework? (4) What environmental, food-related, and socioeconomic risk factors have been quantitatively assessed in association with *Cryptosporidium* infection or detection in the region?

### Eligibility Criteria

Studies were included if they reported primary data on *Cryptosporidium* in human or animal populations within one or more of the 11 Southeast Asian countries: Brunei, Cambodia, Indonesia, Laos, Malaysia, Myanmar, the Philippines, Singapore, Thailand, Timor-Leste, and Vietnam. Eligible studies had to provide information on prevalence, diagnostic methods, or risk factors related to environmental, food, or socioeconomic domains. Only English-language publications were considered.

Studies were excluded if they were not primary research (eg, reviews, editorials, letters, or conference abstracts), were conducted outside the defined geographic region, or did not report on *Cryptosporidium* in human, animal, or environmental samples.

### Information Sources and Search Strategy

We searched five databases: PubMed, Embase, CABI Digital Library, Cochrane Library, and Index Medicus for the South-East Asia Region (IMSEAR). The strategy combined organism-specific terms (eg, “Cryptosporidium,” “Cryptosporidiosis,” “C. parvum,” “C. hominis”) with geographic terms (eg, country names and “Southeast Asia,” “ASEAN”). Searches were designed to maximize inclusivity and minimize missed studies. Full search strategies for each database are provided in Multimedia Appendix 1, reported in accordance with PRISMA-S [34].

Backward citation tracking was performed by reviewing reference lists of included studies and relevant reviews. Targeted searches of organizational websites (eg. World Health Organization, WHO, Food and Agriculture Organization of the United Nations, FAO) were also conducted. No formal gray literature search was performed.

### Screening and Selection Process

All search results were imported into EndNote 21 (Clarivate, Philadelphia, PA) for deduplication. Title and abstract screening, followed by full-text review, was conducted in the Covidence Systematic Review Software (Veritas Health Innovation, Melbourne, Australia) using predefined inclusion and exclusion criteria. Each article was independently screened by two reviewers, with discrepancies resolved by a third reviewer.

### Data Extraction

Data extraction was performed by a single reviewer using a standardized form developed in Covidence. Due to the large number of included studies, dual extraction was not feasible within a reasonable timeframe. To ensure consistency, the form was piloted on ten records, revised based on identified inconsistencies, and the ten piloted records were re-extracted in full before proceeding with the remainder of the dataset. Extracted variables included study metadata (authors, year, country), study design and setting, host population (human, animal, environmental, or mixed), sample type and size, diagnostic methods, species/genotypes, prevalence, study limitations, and reported environmental, food-related, and socioeconomic risk factors. The data dictionary is provided in Multimedia Appendix 2 and is available through OSF (Open Science Framework).

### Data Synthesis

A descriptive synthesis will summarize findings across the included studies. Data will be organized thematically across four domains corresponding to the research questions outlined above: prevalence distribution, diagnostic methods and species resolution, One Health domain integration, and transmission risk factors. Findings will be presented in tables, charts, and maps to illustrate trends in publication volume, geographic distribution, diagnostic approaches, prevalence estimates, species and genotype distribution, and associated risk factors. Descriptive statistics and visualizations will be generated using RStudio (version 2023.09.1+494; R Foundation for Statistical Computing).

Findings will be interpreted with attention to the One Health domain from which data were derived. Prevalence estimates from human, animal, and environmental studies reflect structurally different sampling frames and diagnostic contexts and will not be aggregated across domains. Studies using microscopy-only methods and those using genotyping-based methods yield epidemiologically non-comparable outputs and will be analyzed and presented separately where relevant, as species-level identification is required to distinguish anthroponotic from zoonotic transmission pathways.

No meta-analysis will be conducted. The heterogeneity of diagnostic methods, study populations, and case definitions across the included literature precludes meaningful pooling of prevalence estimates, and formal quantitative synthesis is inconsistent with scoping review methodology as outlined by the Joanna Briggs Institute [32]. No formal risk-of-bias assessment will be performed, consistent with the scoping review methodology.

### Ethics and Dissemination

Results will be disseminated through a peer-reviewed publication targeting journals in the fields of neglected tropical diseases and parasitology. All extracted data and synthesis outputs will be made available in CSV format on the OSF project page [35] in accordance with FAIR (Findable, Accessible, Interoperable, Reusable) principles. No embargo is planned.

## Results

Database searches were conducted from February to September 2024, with the final search completed on September 30, 2024. A total of 889 records were retrieved across five databases prior to deduplication. Following the removal of 177 duplicates, 711 unique records underwent title and abstract screening. Of 333 full-text articles assessed for eligibility, 176 studies were included in the final synthesis, representing nine of eleven Southeast Asian countries and spanning the years 1985 to 2024. Brunei and Timor-Leste had no indexed studies identified in any of the databases searched.

Data extraction and descriptive analysis are complete. The manuscript reporting full findings is in preparation for submission to a peer-reviewed journal.

## Discussion

### Principal Findings

This scoping review will provide a comprehensive synthesis of literature on *Cryptosporidium* in Southeast Asia, mapping research across human, animal, and environmental domains. It will identify geographic and thematic gaps, examine diagnostic practices, and assess the degree of One Health integration. These anticipated findings would extend prior regional syntheses that identified Southeast Asia as a high-priority context for *Cryptosporidium* burden but did not examine diagnostic heterogeneity, One Health integration, or surveillance utility across the regional literature [13,28].

Special attention will be given to diagnostic diversity, including reliance on microscopy versus molecular techniques such as polymerase chain reaction and sequencing, which enables species- and genotype-level identification critical for understanding zoonotic potential. The review will also characterize environmental surveillance efforts, including studies on water and food contamination, and highlight gaps in monitoring systems.

Findings will inform research priorities, support integrated surveillance strategies, and encourage multidisciplinary collaboration under the One Health framework. All extracted data and synthesis outputs will be shared publicly on the OSF in accordance with FAIR principles, ensuring transparency and enabling future meta-analyses and regional comparisons.

Strengths of this review include the pre-registered search strategy, dual-reviewer screening, and cross-domain scope encompassing human, animal, and environmental studies within a single synthesis. Limitations include restriction to English-language publications and single-reviewer data extraction, the latter mitigated by use of a structured extraction form piloted on ten records before full extraction.

### Conclusion

This scoping review will provide a systematic, cross-domain evidence base to support more coordinated and interdisciplinary research on *Cryptosporidium* in Southeast Asia. The findings aim to inform future surveillance strategies, strengthen regional capacity for zoonotic disease prevention, and advance One Health approaches that address the complex drivers of transmission. Ultimately, this work supports the development of context-specific interventions and contributes to broader efforts in health systems resilience.

## Supplementary material

10.2196/89819 Multimedia Appendix 1 Search strategy.

10.2196/89819 Multimedia Appendix 2 Data dictionary.

10.2196/89819 Checklist 1 PRISMA-S checklist.

10.2196/89819 Checklist 2 PRISMA-ScR checklist.

## Abbreviations

ASEAN: Association of Southeast Asian Nations
FAIR: Findable, Accessible, Interoperable, Reusable
FAO: Food and Agriculture Organization of the United Nations
GEMs: Global Enteric Multicenter Study
HIV/AIDS: Human Immunodeficiency Virus / Acquired Immunodeficiency Syndrome
IMSEAR: Index Medicus for the South-East Asia Region
OSF: Open Science Framework
PRISMA-S: Preferred Reporting Items for Systematic Reviews and Meta-Analyses extension for Reporting of Systematic Searches
PRISMA-ScR: Preferred Reporting Items for Systematic Reviews and Meta-Analyses Extension for Scoping Reviews
WHO: World Health Organization
