# Supplementary material for: Mapping Prevalence, Diagnostics, and Evidence Gaps of Cryptosporidium in Southeast Asia Across Human, Animal, and Environmental Domains: Protocol for a One Health Scoping Review
Source: JMIR Res Protoc. 2026 Jun 19;15:e89819. doi: 10.2196/89819 (PMC13282039; doi:10.2196/89819)
Supplement: Multimedia Appendix 1 [file resprot-v15-e89819-s001.docx]

# Multimedia Appendix -Search Strategy

**Reporting note:** The search strategy is reported in accordance with the Preferred Reporting Items for Systematic Reviews and Meta-Analyses extension for Reporting of Systematic Searches (PRISMA-S) [34]. All five databases were searched without date limits or language filters at the database level. An English-language restriction was applied during title and abstract screening. All searches were rerun in full on September 30, 2024, the final search date for all databases.

**Database 1: PubMed** Platform: National Library of Medicine (<https://pubmed.ncbi.nlm.nih.gov>) Date last searched: September 30, 2024, Date limits: None Language limits: None applied at database level

Concept 1: *Cryptosporidium* (organism)

Keywords:

“Cryptosporidium”[tw] OR “Cryptosporidiosis”[tw] OR “cryptosporidia”[tw] OR “C. parvum”[tw] OR “C. hominis”[tw] OR “Cryptosporidium spp”[tw] OR “C. bovis”[tw] OR “C. muris”[tw] OR “C. meleagridis”[tw] OR “C. andersoni”[tw]

MeSH terms:

(“Cryptosporidium”[Mesh]) OR “Cryptosporidiosis”[Mesh] OR “Cryptosporidium parvum”[Mesh]

Combined Concept 1:

(MeSH terms) OR (Keywords)

Concept 2: Geography

Keywords:

Thailand[tw] OR Vietnam[tw] OR Malaysia[tw] OR Indonesia[tw] OR Philippines[tw] OR Laos[tw] OR “Lao People’s Democratic Republic”[tw] OR Cambodia[tw] OR Myanmar[tw] OR Burma[tw] OR Singapore[tw] OR Brunei[tw] OR “Brunei Darussalam”[tw] OR “East Timor”[tw] OR “Timor-Leste”[tw] OR “Southeast Asia”[tw] OR ASEAN

Combined search:

Concept 1 (combined) AND Concept 2 (keywords)

**Database 2: Embase** Platform: Elsevier (<https://www.embase.com>) Date last searched: September 30, 2024, Date limits: None Language limits: None applied at database level

Concept 1: *Cryptosporidium* (organism)

exp Cryptosporidium bovis/ or exp Cryptosporidium parvum/ or exp Cryptosporidium muris/ or exp Cryptosporidium meleagridis/ or exp Cryptosporidium andersoni/ or exp Cryptosporidium hominis/ or exp Cryptosporidium/ or exp Cryptosporidium baileyi/

Concept 2: Geography

(Thailand or Vietnam or Malaysia or Indonesia or Philippines or Laos or Lao People's Democratic Republic or Cambodia or Myanmar or Burma or Singapore or Brunei or Brunei Darussalam or East Timor or Timor-Leste or Southeast Asia or ASEAN).af.

Combined search:

Concept 1 AND Concept 2

**Database 3: CABI Digital Library** Platform: CABI (<https://www.cabidigitallibrary.org>) Date last searched: September 30, 2024, Date limits: None Language limits: None applied at database level

Concept 1: *Cryptosporidium* (organism)

"Cryptosporidium" OR "Cryptosporidiosis" OR "cryptosporidia" OR "C. parvum" OR "C. hominis" OR "Cryptosporidium spp" OR "C. bovis" OR "C. muris" OR "C. meleagridis" OR "C. andersoni"

Concept 2: Geography

Thailand OR Vietnam OR Malaysia OR Indonesia OR Philippines OR Laos OR "Lao People's Democratic Republic" OR Cambodia OR Myanmar OR Burma OR Singapore OR Brunei OR "Brunei Darussalam" OR "East Timor" OR "Timor-Leste" OR "Southeast Asia" OR ASEAN

Combined search:

Concept 1 AND Concept 2

**Database 4: Cochrane Library** Platform: Wiley (<https://www.cochranelibrary.com>) Date last searched: September 30, 2024, Date limits: None Language limits: None applied at database level

Concept 1: *Cryptosporidium* (organism)

Cryptosporidium

Concept 2: Geography

Thailand OR Vietnam OR Malaysia OR Indonesia OR Philippines OR Laos OR "Lao People's Democratic Republic" OR Cambodia OR Myanmar OR Burma OR Singapore OR Brunei OR "Brunei Darussalam" OR "East Timor" OR "Timor-Leste" OR "Southeast Asia" OR ASEAN

Combined search:

Cryptosporidium AND (Thailand OR Vietnam OR Malaysia OR Indonesia OR Philippines OR Laos OR "Lao People's Democratic Republic" OR Cambodia OR Myanmar OR Burma OR Singapore OR Brunei OR "Brunei Darussalam" OR "East Timor" OR "Timor-Leste" OR "Southeast Asia" OR ASEAN)

**Database 5: IMSEAR (Index Medicus for the South-East Asia Region)** Platform: WHO SEARO (*https://www.globalindexmedicus.net/biblioteca/imsear/*) Date last searched: September 30, 2024, Date limits: None Language limits: None applied at database level

Search:

*Cryptosporidium*

Supplementary searches

Backward citation tracking was performed by reviewing the reference lists of all 176 included studies and of relevant reviews identified during screening. Targeted searches of organizational websites, including the World Health Organization (<https://www.who.int>) and the Food and Agriculture Organization of the United Nations (<https://www.fao.org>), were conducted using the term "Cryptosporidium" combined with Southeast Asian country names. No formal gray literature search was conducted.
