# Supplementary material for: Mapping Prevalence, Diagnostics, and Evidence Gaps of Cryptosporidium in Southeast Asia Across Human, Animal, and Environmental Domains: Protocol for a One Health Scoping Review
Source: JMIR Res Protoc. 2026 Jun 19;15:e89819. doi: 10.2196/89819 (PMC13282039; doi:10.2196/89819)
Supplement: Multimedia Appendix 2 [file resprot-v15-e89819-s002.docx]

Multimedia Appendix – Data Dictionary

| Variable Name | Description | Type | Allowed Values / Format |
| --- | --- | --- | --- |
| Title | Title of the study | String | Free text |
| Authors | Authors of the study | String | Free text |
| Year_Published | Year the study was published | Integer | 1980–2025 |
| Country | Country where the study was conducted | String | Brunei, Cambodia, Indonesia, Laos, Malaysia, Myanmar, Philippines, Singapore, Thailand, Timor-Leste, Vietnam |
