# Supplementary material for: Mapping Prevalence, Diagnostics, and Evidence Gaps of Cryptosporidium in Southeast Asia Across Human, Animal, and Environmental Domains: Protocol for a One Health Scoping Review
Source: JMIR Res Protoc. 2026 Jun 19;15:e89819. doi: 10.2196/89819 (PMC13282039; doi:10.2196/89819)
Supplement: Checklist 1 [file resprot-v15-e89819-s003.docx]

**Multimedia Appendix - PRISMA-S Checklist**

| **Section/topic** | **#** | **Checklist item** | **Location(s) Reported** |
| --- | --- | --- | --- |
| **INFORMATION SOURCES AND METHODS** | | | |
| Database name | 1 | Name each individual database searched, stating the platform for each. | Multimedia Appendix (Search Strategy): Database 1: PubMed — National Library of Medicine (https://pubmed.ncbi.nlm.nih.gov); Database 2: Embase — Elsevier (https://www.embase.com); Database 3: CABI Digital Library — CABI (https://www.cabidigitallibrary.org); Database 4: Cochrane Library — Wiley (https://www.cochranelibrary.com); Database 5: IMSEAR — WHO SEARO (https://www.globalindexmedicus.net/biblioteca/imsear/) |
| Multi-database searching | 2 | If databases were searched simultaneously on a single platform, state the name of the platform, listing all of the databases searched. | Not applicable. Each database was searched on its own platform. |
| Study registries | 3 | List any study registries searched. | Not applicable. No study registries were searched. |
| Online resources and browsing | 4 | Describe any online or print source purposefully searched or browsed (e.g., tables of contents, print conference proceedings, web sites), and how this was done. | Methods (Information Sources and Search Strategy, paragraph 2); Multimedia Appendix (Search Strategy, Supplementary searches): WHO (https://www.who.int) and FAO (https://www.fao.org) websites searched using Cryptosporidium combined with Southeast Asian country names. |
| Citation searching | 5 | Indicate whether cited references or citing references were examined, and describe any methods used for locating cited/citing references (e.g., browsing reference lists, using a citation index, setting up email alerts for references citing included studies). | Methods (Information Sources and Search Strategy, paragraph 2); Multimedia Appendix (Search Strategy, Supplementary searches): Reference lists of all 176 included studies and relevant reviews identified during screening were examined. |
| Contacts | 6 | Indicate whether additional studies or data were sought by contacting authors, experts, manufacturers, or others. | Not applicable. No authors, experts, or manufacturers were contacted. |
| Other methods | 7 | Describe any additional information sources or search methods used. | Not applicable. No additional search methods were used beyond those described. |
| **SEARCH STRATEGIES** | | | |
| Full search strategies | 8 | Include the search strategies for each database and information source, copied and pasted exactly as run. | Multimedia Appendix (Search Strategy): Full search strategies provided for all five databases as executed, including platform, date last searched, and complete search syntax. |
| Limits and restrictions | 9 | Specify that no limits were used, or describe any limits or restrictions applied to a search (e.g., date or time period, language, study design) and provide justification for their use. | Multimedia Appendix (Search Strategy, Reporting note): No date limits or language filters were applied at the database level. English-language restriction was applied during title and abstract screening. |
| Search filters | 10 | Indicate whether published search filters were used (as originally designed or modified), and if so, cite the filter(s) used. | Not applicable. No published search filters were used. |
| Prior work | 11 | Indicate when search strategies from other literature reviews were adapted or reused for a substantive part or all of the search, citing the previous review(s). | Not applicable. Search strategies from other reviews were not adapted or reused. |
| Updates | 12 | Report the methods used to update the search(es) (e.g., rerunning searches, email alerts). | Multimedia Appendix (Search Strategy, Reporting note): All searches were rerun in full on September 30, 2024. |
| Dates of searches | 13 | For each search strategy, provide the date when the last search occurred. | Multimedia Appendix (Search Strategy, Reporting note): All five databases were last searched on September 30, 2024. |
| **PEER REVIEW** | | | |
| Peer review | 14 | Describe any search peer review process. | Methods (Information Sources and Search Strategy): Search strategy developed with guidance from a health sciences librarian (Gary Atwood, Dana Health Sciences Library, University of Vermont). No formal peer review of the search strategy was conducted beyond librarian consultation. |
| **MANAGING RECORDS** | | | |
| Total Records | 15 | Document the total number of records identified from each database and other information sources. | Results: 889 total records retrieved across five databases prior to deduplication. Breakdown by individual database not available. |
| Deduplication | 16 | Describe the processes and any software used to deduplicate records from multiple database searches and other information sources. | Methods (Screening and Selection Process): Deduplication performed in EndNote 21 (Clarivate, Philadelphia, PA). 177 duplicates removed (176 identified in EndNote; 1 identified during Covidence screening), yielding 711 unique records. |
|  |  |  |  |
| PRISMA-S: An Extension to the PRISMA Statement for Reporting Literature Searches in Systematic Reviews | | |  |
| Rethlefsen ML, Kirtley S, Waffenschmidt S, Ayala AP, Moher D, Page MJ, Koffel JB, PRISMA-S Group. | | |  |
| Last updated February 27, 2020. | |  |  |
