# Supplementary material for: Mapping Prevalence, Diagnostics, and Evidence Gaps of Cryptosporidium in Southeast Asia Across Human, Animal, and Environmental Domains: Protocol for a One Health Scoping Review
Source: JMIR Res Protoc. 2026 Jun 19;15:e89819. doi: 10.2196/89819 (PMC13282039; doi:10.2196/89819)
Supplement: Checklist 2 [file resprot-v15-e89819-s004.docx]

**Multimedia Appendix - Preferred Reporting Items for Systematic reviews and Meta-Analyses extension for Scoping Reviews (PRISMA-ScR) Checklist**

| **SECTION** | **ITEM** | **PRISMA-ScR CHECKLIST ITEM** | **REPORTED ON PAGE #** |
| --- | --- | --- | --- |
| **TITLE** | | | |
| Title | 1 | Title | 1 |
| **ABSTRACT** | | | |
| Structured summary | 2 | Abstract (all structured sections: Background, Objective, Methods, Results, Conclusions, Trial Registration) | 2-3 |
| **INTRODUCTION** | | | |
| Rationale | 3 | Introduction, paragraphs 1-4 | 4-5 |
| Objectives | 4 | Introduction, paragraph 5 (research questions); Methods, Objectives subsection | 5-6 |
| **METHODS** | | | |
| Protocol and registration | 5 | Methods, Study Design and Framework. Protocol registered on OSF; DOI: 10.17605/OSF.IO/QV27Y | 7 |
| Eligibility criteria | 6 | Methods, Eligibility Criteria | 7-8 |
| Information sources* | 7 | Methods, Information Sources and Search Strategy; Multimedia Appendix, Search Strategy | 8, appendix |
| Search | 8 | Multimedia Appendix, Search Strategy (full syntax provided for all five databases) | 8, appendix |
| Selection of sources of evidence† | 9 | Methods, Screening and Selection Process | 8-9 |
| Data charting process‡ | 10 | Methods, Data Extraction | 9 |
| Data items | 11 | Methods, Data Extraction; Multimedia Appendix, Data Dictionary | 9, appendix |
| Critical appraisal of individual sources of evidence§ | 12 | Not applicable. No formal critical appraisal was conducted, consistent with scoping review methodology. | N/A |
| Synthesis of results | 13 | Methods, Data Synthesis | 9-10 |
| **RESULTS** | | | |
| Selection of sources of evidence | 14 | Results. 889 records retrieved; 177 duplicates removed; 711 screened; 333 full-text assessed; 176 included. PRISMA flow diagram will be reported in the full scoping review manuscript. | 10 |
| Characteristics of sources of evidence | 15 | Not applicable at protocol stage. Characteristics of included sources will be reported in the full scoping review manuscript. | N/A |
| Critical appraisal within sources of evidence | 16 | Not applicable. No critical appraisal was conducted (see Item 12). | N/A |
| Results of individual sources of evidence | 17 | Not applicable at protocol stage. Results for individual sources will be reported in the full scoping review manuscript. | N/A |
| Synthesis of results | 18 | Not applicable at protocol stage. Synthesis results will be reported in the full scoping review manuscript. | N/A |
| **DISCUSSION** | | | |
| Summary of evidence | 19 | Discussion, paragraph 1 | 11 |
| Limitations | 20 | Discussion, Strengths and Limitations paragraph | 11 |
| Conclusions | 21 | Conclusion | 11 |
| **FUNDING** | | | |
| Funding | 22 | Funding. This study received no specific funding from any public, commercial, or not-for-profit funding agencies. | 12 |

JBI = Joanna Briggs Institute; PRISMA-ScR = Preferred Reporting Items for Systematic reviews and Meta-Analyses extension for Scoping Reviews.

* Where *sources of evidence* (see second footnote) are compiled from, such as bibliographic databases, social media platforms, and Web sites.

† A more inclusive/heterogeneous term used to account for the different types of evidence or data sources (e.g., quantitative and/or qualitative research, expert opinion, and policy documents) that may be eligible in a scoping review as opposed to only studies. This is not to be confused with *information sources* (see first footnote).

‡ The frameworks by Arksey and O’Malley (6) and Levac and colleagues (7) and the JBI guidance (4, 5) refer to the process of data extraction in a scoping review as data charting*.*

§ The process of systematically examining research evidence to assess its validity, results, and relevance before using it to inform a decision. This term is used for items 12 and 19 instead of "risk of bias" (which is more applicable to systematic reviews of interventions) to include and acknowledge the various sources of evidence that may be used in a scoping review (e.g., quantitative and/or qualitative research, expert opinion, and policy document).

*From:* Tricco AC, Lillie E, Zarin W, O'Brien KK, Colquhoun H, Levac D, et al. PRISMA Extension for Scoping Reviews (PRISMAScR): Checklist and Explanation. Ann Intern Med. 2018;169:467–473. [doi: 10.7326/M18-0850](http://annals.org/aim/fullarticle/2700389/prisma-extension-scoping-reviews-prisma-scr-checklist-explanation).
